# Cerebral blood flow quantification with multi-delay arterial spin labeling in ischemic stroke and the association with early neurological outcome

**DOI:** 10.1016/j.nicl.2023.103340

**Published:** 2023-01-31

**Authors:** Sven P.R. Luijten, Daniel Bos, Pieter-Jan van Doormaal, Mayank Goyal, Rick M. Dijkhuizen, Diederik W.J. Dippel, Bob Roozenbeek, Aad van der Lugt, Esther A.H. Warnert

**Affiliations:** a Department of Radiology & Nuclear Medicine, Erasmus MC University Medical Center, the Netherlands; b Department of Radiology, Foothills Medical Center, University of Calgary, Canada; c Biomedical MR Imaging and Spectroscopy Group, Center for Image Sciences, University Medical Center Utrecht & Utrecht University, the Netherlands; d Department of Neurology, Erasmus MC University Medical Center, the Netherlands

**Keywords:** Arterial spin labeling, Cerebral blood flow, Hyperperfusion, Large vessel occlusion, Ischemic stroke, LVO, large vessel occlusion, EVT, endovascular thrombectomy, ASL, arterial spin labeling, PLD, post-labeling delay, ATT, arterial transit time, NIHSS, National Institutes of Health Stroke Scale, AOL, arterial occlusive lesion

## Abstract

Restoring blood flow to brain tissue at risk of infarction is essential for tissue survival and clinical outcome. We used cerebral blood flow (CBF) quantified with multiple post-labeling delay (PLD) pseudocontinuous arterial spin labeling (ASL) MRI after ischemic stroke and assessed the association between CBF and early neurological outcome. We acquired ASL with 7 PLDs at 3.0 T in large vessel occlusion stroke patients at 24 h. We quantified CBF relative to the contralateral hemisphere (rCBF) and defined hyperperfusion as a ≥30% increase and hypoperfusion as a ≥40% decrease in rCBF. We included 44 patients (median age: 70 years, median NIHSS: 13, 40 treated with endovascular thrombectomy) of whom 37 were recanalized. Hyperperfusion in ischemic core occurred in recanalized but not in non-recanalized patients (65.8% vs 0%, p = 0.006). Hypoperfusion occurred only in the latter group (0% vs 85.7%, p < 0.001). In recanalized patients, hyperperfusion was also seen in salvaged penumbra (38.9%). Higher rCBF in ischemic core (aβ, −2.75 [95% CI: −4.11 to −1.40]) and salvaged penumbra (aβ, −5.62 [95% CI: −9.57 to −1.68]) was associated with lower NIHSS scores at 24 h. In conclusion, hyperperfusion frequently occurs in infarcted and salvaged brain tissue following successful recanalization and early neurological outcome is positively associated with the level of reperfusion.

## Highlights

• Multi-PLD ASL allows quantification of cerebral blood flow in ischemic stroke.
• Hyperperfusion in ischemic regions is common after recanalization with EVT.
• Early neurological outcome is positively associated with the level of reperfusion.

## 1 Introduction

In patients presenting with ischemic stroke due to large vessel occlusion (LVO), timely intervention to remove the occluding thrombus is pivotal and can be accomplished by endovascular thrombectomy (EVT) with or without intravenous thrombolysis (Goyal et al., 2016). The success of this treatment strategy is largely attributed to the extent of recanalization after the intervention, which can be assessed using imaging modalities capable of visualizing the vessel flow such as digital subtraction angiography (DSA), computed tomography angiography (CTA), and magnetic resonance angiography (MRA) (Liebeskind et al., 2019). However, the degree of reperfusion i.e., the restoration of blood flow more distally in brain tissue at risk of infarction due to LVO-related decrease in perfusion, is a more accurate indicator of tissue survival and clinical outcome (Soares et al., 2010, Eilaghi et al., 2013).

ASL allows for the possibility to non-invasively visualize and quantify cerebral blood flow (CBF) (Haller et al., 2016). Recently, several studies have used ASL to quantify CBF with the aim to monitor success of EVT and predict neurological outcomes (Yu et al., 2015, Yoo et al., 2018, Shimonaga et al., 2019, Lu et al., 2021; Ter Schiphorst et al., 2021). Often these studies acquired ASL with a single delay period between labeling of blood in the feeding cervical arteries and image acquisition to detect inflow of labeled blood in the brain. However, use of such single post-labeling delay (PLD) protocols does not consider variation in the timing of arrival of labeled blood, i.e. the arterial transit time (ATT), between different brain regions when quantifying CBF (Woods et al., 2019, Woods et al., 2020). Single-PLD ASL could potentially be suboptimal in ischemic stroke patients, as ATT is likely to vary between affected and unaffected brain regions due to altered hemodynamics after recanalization with EVT (Kneihsl et al., 2018, Zhang et al., 2019). Acquiring ASL with multiple PLDs and proper kinetic modeling mitigates this issue by providing dynamic assessment of labeled blood inflow making it possible to quantify both ATT and CBF taking into account the variability of ATT among different brain regions (Dai et al., 2013).

In the present study, we quantified CBF in ischemic core and salvaged penumbra in patients after ischemic stroke due to LVO and assessed the association between CBF and early neurological outcome. Moreover, we investigated the difference between using multi-PLD versus single-PLD ASL for CBF quantification. CBF was therefore calculated using 1) multi-PLD ASL with correction of ATT and 2) using images acquired at a single-PLD to model CBF based on fixed ATT.

## 2 Materials and methods

### 2.1 Study population

For this study, we used data from ischemic stroke patients who were included in the MR CLEAN MED, MR CLEAN NO-IV, or MR CLEAN LATE trial (van der Steen et al., 2022, LeCouffe et al., 2021, Pirson et al., 2021) at a single EVT capable intervention center (Erasmus University Medical Center) between October 2019 and November 2021 (supplement Fig. 1). All trials included patients 18 years or older, with anterior LVO including intracranial carotid artery (ICA), ICA terminus (ICA-T), or M1/M2 middle cerebral artery (MCA) segment occlusion, and a National Institutes of Health Stroke Scale (NIHSS) score ≥ 2. Patients presenting within 4.5 h after stroke onset or last seen well were eligible for inclusion in MR CLEAN NO-IV and patiens presenting within 6 h after stroke onset or last seen well were eligible for the inclusion in MR CLEAN MED. Patients presenting between 6 and 24 h after stroke onset or last seen well were eligible for inclusion in MR CLEAN LATE. For radiological outcome assessment, patients underwent follow-up imaging with either CT or MRI at 24 h. For the current analysis we selected patients with availability of MRI including multi-PLD ASL at 24 h follow-up. Exclusion criteria were ICA or MCA stenosis, parenchymal hematoma type 1 or 2 (von Kummer et al., 2015), bilateral (sub)acute infarction, and motion artefacts during MRI (supplement Fig. 1). Neurological deficit was assessed using the NIHSS at baseline and at 24 h follow-up.

The central ethics committee at Erasmus University Medical Center approved the study protocol of each trial. Each trial was conducted in accordance with the principles of the Declaration of Helsinki (2013), the ICH-GCP principles, and in accordance with the Medical Research Involving Human Subjects Act (WMO). All patients or their legal representatives provided written deferred consent for use of all patient clinical and imaging data.

### 2.2 CT acquisition and analysis

Patients underwent baseline non-contrast CT, CT angiography (CTA), and CT perfusion (CTP) imaging at presentation to the emergency department according to local stroke imaging protocols. Intracranial occlusion location (ICA, ICA-T, M1, M2) was evaluated on CTA imaging by expert neuroradiologists blinded for outcomes. CTP images were centrally analyzed using CT Neuroperfusion software (Syngo.via VB60A, Siemens Healthineers, Germany). Image processing included semi-automatic motion correction, bone removal, brain segmentation, and reference vessel and arterial input function detection using default clinical workflow settings. CTP parameter maps were calculated using a deconvolution-based algorithm (Abels et al., 2010).

### 2.3 MRI acquisition and analysis

Patients underwent follow-up imaging at 24 h after admission to the hospital with 3.0 T MRI (Discovery MR750 or SIGNA Premier, GE Healthcare, Waukesha, USA). We performed multi-PLD 3D pseudocontinuous ASL (TR: 8248 ms, TE: 11 ms, voxel size: 1.9 × 1.9 × 3.5 mm^3^, scan duration: 5 min 41 s) based on Hadamard-encoding with 7 different PLDs (0.8, 1.1, 1.4, 1.8, 2.2, 2.8, 3.5 s) and effective labeling durations (0.23, 0.32, 0.40, 0.52, 0.77, 1.49 s) (Dai et al., 2013, Cohen et al., 2020). Additional scans we acquired included diffusion weighted imaging (DWI) (TR: 8000 ms, TE: 58 ms, voxel size: 0.94 × 0.94 × 3 mm^3^, b-values: 0 and 1000 s/mm^2^, scan duration: 1 min 20 s), 3D fluid-attenuated inversion recovery (FLAIR) (TR: 5002 ms, TE: 92 ms: voxel size: 0.47 × 0.47 × 0.79 mm^3^, scan duration: 4 min 03 s), and 3D time-of-flight (TOF) MR angiography (MRA) (TR: 24 ms, TE: 3 ms, voxel size: 0.43 × 0.43 × 0.40 mm^3^, scan duration: 4 min 36 s).

Recanalization was assessed by expert neuroradiologists blinded for outcomes on 3D TOF-MRA at 24 h using the arterial occlusive lesion (AOL) score (Khatri et al., 2005). Patients with an AOL score of 0–1 were considered non-recanalized and with a score of 2–3 as recanalized (Zaidat et al., 2013). In patients undergoing EVT, recanalization status was also assessed on post-procedural digital subtraction angiography (DSA) images using the expanded Treatment in Cerebral Infarction (eTICI) score. Successful recanalization on post-procedural DSA was considered as eTICI 2B-3.

We used the Bayesian Inference for Arterial Spin Labeling MRI (BASIL) toolkit implemented in FSL (version 6.0.5, Oxford, UK) for analyzing ASL data. This method allows fitting a two compartment model estimating both the tissue and macrovascular contribution to the perfusion signal separately generating CBF and ATT maps (Groves et al., 2009, Chappell et al., 2010). CBF maps were calibrated using a voxel-wise calibration approach (Pinto et al., 2020). In order to compare quantification of CBF when using 7 PLDs versus only a single-PLD, we also quantified CBF using the perfusion images acquired with a PLD of 1.8 s. The latter analysis was done using a standard ATT of gray matter (GM) of 1.3 s (Juttukonda et al., 2021). All analyses were done in native ASL space.

We assessed CBF and ATT in the following regions of interest (ROIs): 1) Ischemic core characterized as hyperintensity due to reduced tissue water diffusion was manually delineated on DWI b1000 at 24 h using ITK-SNAP (http://www.itksnap.org/) and visually confirmed as hypointensity on apparent diffusion coefficient (ADC) maps. ADC maps were calculated from the DWI acquisition using vendor specific software (Functool, GE Healthcare); 2) Salvaged penumbra defined as tissue with a time-to-maximum (Tmax) delay of >6 s (Olivot et al., 2009) within the affected hemisphere assessed on baseline CTP minus ischemic core. Under the assumption that penumbral tissue is salvaged in case of successful recanalization and otherwise converted to ischemic core, we only defined salvaged penumbra in recanalized patients (AOL score 2–3); 3) Contralateral normal brain by mirroring the ischemic core to the unaffected hemisphere; and 4) Healthy GM in the unaffected hemisphere determined from the MNI standard atlas. Within each ROI, we included only voxels with a partial volume estimate for GM of >70 % from the MNI standard atlas. In addition, we also assessed the occurrence of hyperperfusion and hypoperfusion in ischemic core and salvaged penumbra. Based on previous literature and for comparison purposes, we defined hyperperfusion as a ≥30% increase and hypoperfusion as a ≥40% decrease in CBF compared to contralateral mirrored brain regions (rCBF) (Bivard et al., 2014, Bhaskar et al., 2017, Lu et al., 2021; Ter Schiphorst et al., 2021). Comparisons with mirrored brain regions were made in order to compare CBF between topographically homologous regions with similar distributions of grey and white matter.

### 2.4 Image registration

Ischemic core masks were registered to ASL space through rigid registration (FLIRT) of DWI b0 to M0 images because of their higher anatomical detail (Jenkinson et al., 2002). GM masks from the MNI standard atlas registered to ASL image space by inverse normalization. In order to do so, we first performed linear registration of M0 images to FLAIR images followed by non-linear registration (FNIRT) of FLAIR images to a FLAIR-based MNI standard atlas in order to match relative signal intensities between the input and reference images (Winkler et al., 2009, Harston et al., 2017). Tissue masks with a Tmax delay >6 s (smoothed 5 mm FWHM) were registered to ASL space by performing rigid registration of CTP images to M0 images using a mutual cost function and without prior brain extraction (BET) as this has been shown to result in more accurate intermodal registration between CT and MRI (Hinds et al., 2018). Thereafter, salvaged tissue masks were computed by subtracting voxels labeled as ischemic core from voxels labeled with a Tmax delay >6 s in native ASL space.

### 2.5 Statistical analysis

Differences between recanalized and non-recanalized patients were assessed using independent samples *t*-test, chi-square test, or Mann-Whitney U for nonparametric testing. Comparisons of mean (±SD) CBF and ATT between different ROIs were made using one-way ANOVA with Tukey-Kramer post-hoc tests. Next, we assessed the association of rCBF in ischemic core and penumbra, as measure of reperfusion, with NIHSS score at 24 h using linear regression. We adjusted only for baseline NIHSS and ischemic core volume on DWI (Mistry et al., 2022). Effect estimates are presented as adjusted beta (aβ) with 95 % confidence interval (CI). Lastly, we used paired t-tests to compare mean CBF values within each ROI derived using 7 PLDs versus using only one PLD (1.8 s). Statistical analyses were performed using R statistical programming.

## 3 Results

We included 44 patients (median age, 70 years [interquartile range: 60–78 years]; 20 women; Table 1) of whom 37 were recanalized. Baseline CTP was not routinely performed by all primary stroke center referring patients for EVT at the Erasmus University Medical Center. Consequently, salvaged penumbra could only be determined in a subset of recanalized patients (18/37, 48.6%). Median time between last seen well and follow-up MRI was 27 h (IQR, 25–36 h) and median time between admission to the hospital and follow-up MRI was 24 h (IQR, 23–28 h). An eTICI 2B-3 on post-procedural DSA was achieved in all recanalized patients and in 1 non-recanalized patient in whom subsequent reocclusion at the same target location was observed on MRA at 24 h (100% vs 14.3%; p < 0.001). Recanalized patients had smaller ischemic core volumes on DWI at 24 h (median [IQR], 10 ml [3–18] vs 49 ml [36–136]; p = 0.001) and lower NIHSS scores at 24 h (median [IQR] 2 [1–5] vs 8 [8–19]; p = 0.001) than non-recanalized patients.

**Table 1:** Patient characteristics.

	**All patients** (n = 44)	**Recanalized** (n = 37)	**Non-recanalized** (n = 7)	**p-value**
**Age**, years	70 (60–78)	69 (60–78)	73 (58–75)	0.71
**Female sex**	20 (45.5%)	16 (43.2%)	4 (57.1%)	0.79
**Baseline NIHSS**	13 (9–20)	13 (8–20)	12 (10–20)	0.55
**IV thrombolysis**	27 (61.4%)	25 (67.6%)	2 (28.6%)	0.13
**EVT**	40 (90.1%)	37 (100%)	3 (42.9%)	<0.001
**Occlusion location**				0.45
ICA/ICA-T	7 (11.4%)	7 (18.9%)	0 (0%)	
M1	22 (56.8%)	18 (48.6%)	4 (57.1%)	
M2	15 (31.8%)	12 (32.4%)	3 (42.9%)	
**Successful recanalization on DSA** (eTICI 2B-3)*	38 (86.4%)	37 (100%)	1 (14.3%)	<0.001
**Time between stroke onset to baseline CT imaging**, minutes	107 (61–367)	84 (59–261)	348 (296–617)	0.06

Values are presented as counts (%) or median (IQR). P-values given for comparisons between recanalized and non-recanalized patients.

*Successful recanalization on DSA could not be assessed in 4 patients who did not undergo EVT.

Abbreviations: NIHSS, National Institutes of Health Stroke Scale; EVT, endovascular thrombectomy; ICA, intracranial internal carotid artery.

### 3.1 CBF and ATT among brain regions

**Fig. 1:**
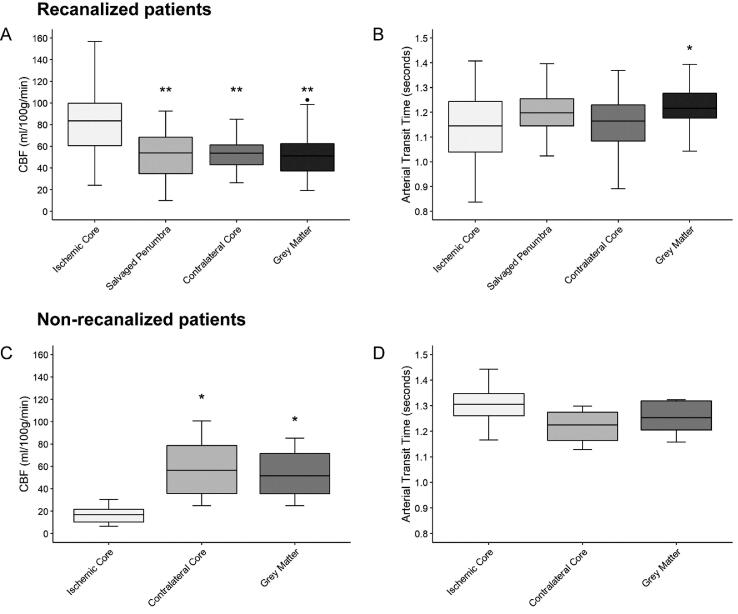
Boxplots showing CBF and ATT values in each ROI stratified by recanalization status. (A) CBF and (B) ATT values in recanalized patients (n = 37). (C) CBF and (D) ATT values in non-recanalized patients (n = 7). Boxplots indicate from top to bottom, the maximum, 75th, 50th, 25th percentiles, and minimum. Outliers are indicated by black dots. Values in salvaged penumbra are derived from a subset of recanalized patients with baseline CTP available (n = 18). *P < 0.05 compared to ischemic core. **P < 0.001 compared to ischemic core.

In recanalized patients, CBF differed between brain ROIs (F_3,125_ = 18.33; p < 0.001). Pairwise comparisons showed significantly higher CBF in ischemic core (84.8 ± 33.0 ml/100 g/min) compared to salvaged penumbra (55.0 ± 20.3 ml/100 g/min), normal brain (53.7 ± 15.1 ml/100 g/min), and healthy GM (49.6 ± 16.8 ml/100 g/min; all p < 0.001; Fig. 1A). ATT in ischemic core showed great variability and was significantly shortened when compared to healthy GM (1.14 ± 0.14 vs 1.22 ± 0.08 s, p = 0.01; Fig. 1B). In non-recanalized patients, CBF also differed between brain ROIs (F_2,18_ = 6.123; p = 0.009). In contrast, CBF was significantly lower in ischemic core (16.5 ± 9.0 ml/100 g/min) compared to normal brain (48.4 ± 23.1 ml/100 g/min, p = 0.01) and healthy GM (44.5 ± 20.6 ml/100 g/min, p = 0.03; Fig. 1C). ATT, on the other hand, was similar across different brain ROIs (F_2,18_ = 0.626; p = 0.55; Fig. 1D).

**Fig. 2:**
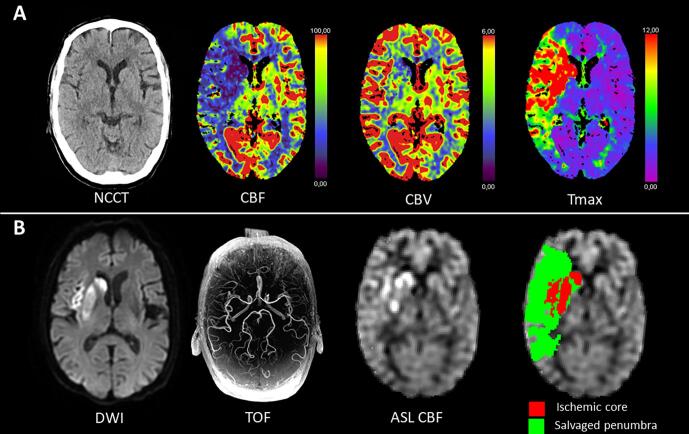
Example images of a patient presenting with a right M1-MCA occlusion and substantial neurological deficit (baseline NIHSS = 11) who was successfully treated with thrombectomy (eTICI 2B). (A) Top row includes baseline CT images showing large perfusion deficit in the right MCA territory with subtle loss of grey-white matter differentiation in the basal ganglia. (B) Follow-up MR imaging at 24 h in bottom row. DWI shows infarction of the basal ganglia and insular cortex with patent right MCA on TOF-MRA. ASL CBF map shows hyperperfusion mainly in ischemic core (red) and also slightly elevated perfusion in salvaged penumbra (green). This patient had excellent early neurological recovery at 24 h (NIHSS = 0).

**Fig. 3:**
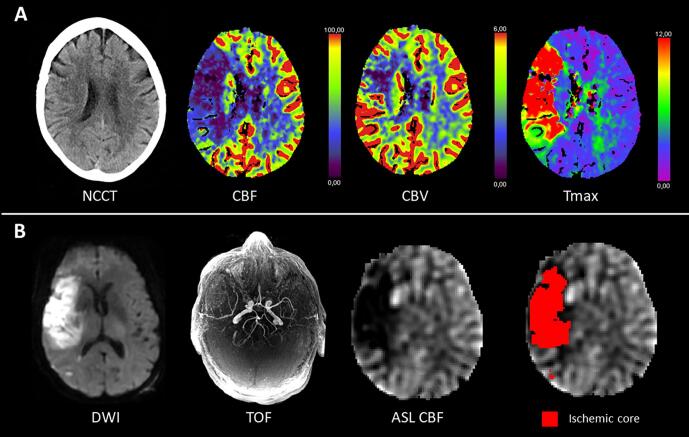
Example images of a patient presenting with a right M1-MCA occlusion and substantial neurological deficit (baseline NIHSS = 12) who was not successfully treated with thrombectomy (eTICI 0). (A) Top row includes baseline CT images showing large perfusion deficit in the right MCA territory. (B) Follow-up MR imaging at 24 h in bottom row. DWI shows a large area of infarction with a persistent occlusion of the right M1-MCA on TOF-MRA. ASL CBF map shows hypoperfusion in the ischemic core (red). This patient’s neurological deficit increased at 24 h (NIHSS = 17).

**Fig. 4:**
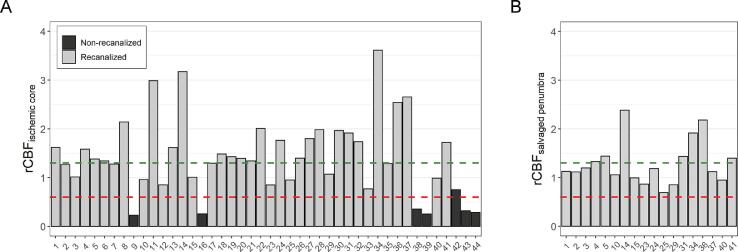
CBF in the ischemic core and salvaged penumbra relative to mirrored contralateral brain regions (rCBF). (A) shows rCBF in ischemic core in all patients (n = 44) stratified by recanalization status. (B) shows rCBF in salvaged penumbra in recanalized patients with baseline CTP available (n = 18). Each bar represents an individual patient and the unique patient numbers are listed on the x-axis. The dashed green line indicates the threshold for hyperperfusion and the dashed red line indicates the threshold for hypoperfusion.

We also compared the occurrence of hyperperfusion (Fig. 2) and hypoperfusion (Fig. 3) between recanalized and non-recanalized patients. This revealed that hyperperfusion in ischemic core occurred in recanalized but not in non-recanalized patients (24/37 [65.8%] vs 0/7 [0%], p = 0.006) while hypoperfusion occurred only in the latter group (0/37 [0%] vs 6/7 [85.7%], p < 0.001; Fig. 4). In addition, in recanalized patients, we found that hyperperfusion also occurred in salvaged penumbra (7/18, 38.9 %; Fig. 4).

### 3.2 Association with early neurological outcome

We found a significant association between rCBF in ischemic core with lower NIHSS scores at 24 h in the total study population (aβ per 100% increase, −2.75 [95% CI: −4.11 to −1.40]; n = 44). When limiting the analysis to recanalized patients, a similar association between rCBF in ischemic core with lower NIHSS at 24 h was found (aβ per 100% increase, −2.42 [95% CI: −4.02 to −0.82]; n = 37). Additionally, in recanalized patients with baseline CTP available, we found a significant association between rCBF in salvaged penumbra with lower NIHSS scores at 24 h (aβ per 100% increase, −5.62 [95% CI: −9.57 to −1.68]; n = 18).

### 3.3 CBF quantification with different PLDs

**Fig. 5:**
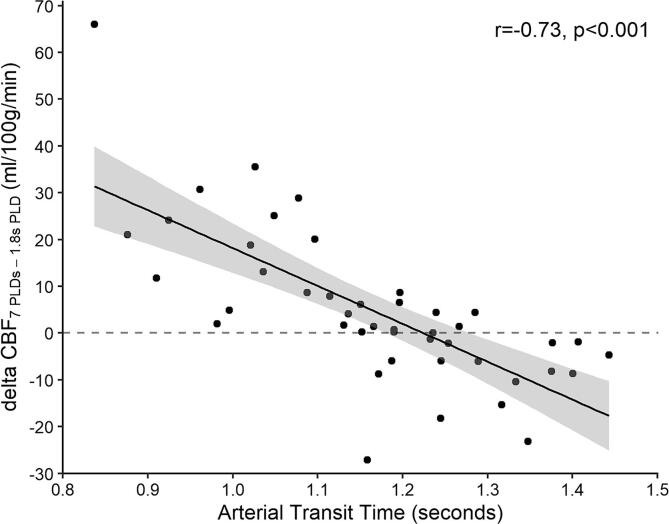
Scatterplot showing difference in CBF quantified using 7 PLDs versus images acquired at a PLD of 1.8 s (delta CBF_7 PLDS – 1.8s PLD_) over arterial transit time in ischemic core (n = 44). Corresponding regression line for linear fit is shown in black with 95% confidence interval in shaded grey. Pearson correlation coefficient shown in top right corner. Dashed grey line indicates no difference between CBF quantified using 7 PLDs versus images acquired at a PLD of 1.8 s.

CBF in ischemic core estimated using 7 PLDs was significantly higher compared to CBF estimated using a single-PLD of 1.8 s (84.8 ± 33.0 vs 78.6 ± 25.0 ml/100 g/min, p = 0.04; supplement Table 1). Comparisons within individual patients showed that differences between CBF values in ischemic core using 7 PLDs versus a single-PLD of 1.8 s increased with shortened ATT (r = -0.73, p < 0.001; Fig. 5). In the other ROIs, similar CBF values were derived from ASL images using 7 PLDs compared to a PLD of 1.8 s (supplement Table 1).

## 4 Discussion

In this study we used multi-PLD ASL to quantify CBF and ATT in patients after ischemic stroke due to LVO. We found that among recanalized patients CBF was significantly higher in ischemic core compared to other brain regions whereas, in non-recanalized patients, CBF was significantly lower in ischemic core. When comparing CBF values within individual recanalized patients, we found that hyperperfusion was seen both in ischemic core and also in salvaged penumbra and that early neurological outcome is positively associated with the level of reperfusion. Conversely, hypoperfusion was seen only in non-recanalized patients. Additionally, we found that using ASL images acquired at a single PLD of 1.8 s led to lower CBF estimates in ischemic core compared to multi-PLD ASL in recanalized patients. Furthermore, we found that shortened ATT within ischemic core correlated with greater differences in CBF estimates between single-PLD and multi-PLD ASL.

Consistent with previous studies, we demonstrate that hyperperfusion can frequently be seen following successful recanalization (Shimonaga et al., 2019, Lu et al., 2021). Adding to previous work, however, we now spatially localized the occurrence of hyperperfusion and hypoperfusion in different ischemic compartments. The finding that hyperperfusion occurred more frequently and to a greater extent in ischemic core compared to salvaged penumbra suggests there is a correlation with the severity of CBF impairment or the degree of ischemic tissue injury prior to recanalization. Data further supporting this hypothesis found that, in brain regions where hyperperfusion was seen following successful recanalization with intra-arterial thrombolysis, ADC values on pretreatment MRI were lower indicative of greater ischemic distress (Kidwell et al., 2001). Hypoperfusion, on the other hand, was seen only in non-recanalized patients. This finding corresponds with a recent study using the same threshold of rCBF reduction (≥40%) and found that hypoperfusion in successfully recanalized patients is rare and occurred only in 1 out of 33 patients (Ter Schiphorst et al., 2021).

The underlying mechanisms of cerebral hyperperfusion following successful recanalization are not well understood. It has been suggested to be caused by a loss of myogenic tone in cerebral arteries leading to vasodilation and as such reflects the loss of cerebral autoregulation rather than increased tissue metabolism (Palomares and Cipolla, 2011). It seems plausible that such loss of cerebral autoregulation is more likely to occur in ischemic core which is considered non-viable tissue. However, in our study, hyperperfusion was also seen in viable salvaged penumbra, albeit less frequent and to a lesser extent. It may thus be worthwhile to complement post-recanalization assessment of CBF with metabolic parameters such as oxygen extraction fraction and cerebral metabolic rate of oxygen (Fan et al., 2020, Wu et al., 2021). These parameters are measures of the extent to which oxygen is extracted from arterial blood and the rate of oxygen consumption, respectively, and thus reflect tissue metabolism (Lin and Powers, 2018). Such an approach could potentially be valuable for helping to make a distinction between hyperperfusion due to loss of cerebral autoregulation as opposed to increased tissue metabolism.

Previous studies have repeatedly found a relation between hyperperfusion on post-recanalization imaging and favorable neurological outcomes (Yoo et al., 2018, Shimonaga et al., 2019, Lu et al., 2021). Correspondingly, we also demonstrate an association of higher rCBF values within the ischemic core and salvaged penumbra with lower NIHSS scores at 24 h. These findings may seem counterintuitive when considering that hyperperfusion is possibly related to the degree of ischemic tissue injury prior to recanalization and reflects the loss of cerebral autoregulation. However, in our study, we demonstrate that hyperperfusion in ischemic core and salvaged penumbra occurred only after successful recanalization which is known to be associated with better neurological recovery. The prognostic value of hyperperfusion may thus be determined by the interplay between hyperperfusion as a measure of the degree of ischemic tissue injury and the quality of recanalization. Also, in recanalized patients, there may be a fine balance between the beneficial effects of increased CBF to viable penumbral tissue and the hazardous effects of increased CBF to severely damaged ischemic core. Furthermore, previous studies have found that the latter is associated with an increased risk of intracranial hemorrhage (ICH) post-recanalization (Yu et al., 2015, Shimonaga et al., 2019). In the current study, however, CBF was assessed at 24 h after ischemic stroke, when ICHs, if present, have often already occurred (Yu et al., 2015). Moreover, patients with parenchymal hematomas were excluded from the study. The causal relationship between hyperperfusion and the occurrence of ICH could therefore not be reliably assessed.

Comparisons of CBF quantification using ASL images acquired with 7 PLDs versus a single-PLD of 1.8 s revealed that the latter provided lower CBF estimates in ischemic core in recanalized patients. This is because CBF quantification using multiple PLDs samples the dynamic inflow of labeled blood. By doing so, we were able to correct for variable ATT between different brain regions which we showed is often shortened in the ischemic core with substantial intersubject variability. Due to the shortened ATT in ischemic core in successfully recanalized patients, a conventional PLD of 1.8 s may actually be suboptimal for estimating CBF (Woods et al., 2020). This is further supported by the observation that individual differences in CBF quantified in ischemic core using ASL images acquired with 7 PLDs versus a PLD of 1.8 s became greater with shorter ATT. In turn, in other brain regions with similar ATT and less variability, we found no differences in CBF quantification with 7 PLDs versus a PLD of 1.8 s. These findings potentially have important clinical implications when using ASL in ischemic stroke patients. Previous studies often used single-PLD ASL to quantify CBF in ischemic territories relative to the contralateral hemisphere to monitor success of EVT and predict neurological outcomes (Yu et al., 2015, Yoo et al., 2018, Lu et al., 2021; Ter Schiphorst et al., 2021). Yet, here we show that single-PLD ASL provided lower CBF estimates in ischemic core compared to multi-PLD ASL, especially in patients with shortened ATT. Multi-PLD ASL is desirable in these cases as it allows for CBF quantification with correction for variation in ATT. In theory, this approach allows for more accurate perfusion quantification and detection of perfusion abnormalities (Zhao et al., 2021). However, we did not compare multi-PLD versus single-PLD CBF quantification against another imaging technique such as dynamic susceptibility contrast MRI or positron emission tomography as reference. Therefore, our study does not provide evidence that multi-PLD ASL is more accurate than single-PLD ASL for quantification of CBF in the present setting. Such a comparison is desirable to determine whether multi-PLD ASL is truly more accurate than single-PLD ASL for quantification of CBF after recanalization. This is also clinically relevant to know if the interplay between the beneficial and adverse effects of hyperperfusion rely on the extent to which CBF is increased.

Limitations of this study that must be considered are its single center design introducing a possible selection bias and limiting the generalizability of our findings. We included a small sample of non-recanalized patients, limiting the ability to detect potential significant differences in this group. Due to the cross-sectional nature of our study, we were not able to investigate the causal relationships of post-recanalization CBF with radiological and neurological outcomes at later time points. Performing ASL imaging immediately after EVT and at later time points is needed to better investigate how perfusion status further develops and to investigate the relationship between post-recanalization changes in CBF and ischemic core growth, occurrence of ICH, and neurological recovery (Franx et al., 2021). Lastly, baseline CTP was not routinely performed by all regional primary stroke centers referring patients for EVT and thus we were not able to determine salvaged penumbral tissue in all recanalized patients. Therefore, larger prospective studies including baseline perfusion imaging are needed to confirm our findings.

In conclusion, multi-PLD ASL can be used to quantify post-ischemic CBF and ATT in ischemic stroke patients. Hyperperfusion frequently occurs both in infarcted and salvaged brain tissue following successful recanalization and early neurological outcome is positively associated with the level of reperfusion. Hypoperfusion, on the other hand, is frequently seen in infarcted brain tissue in case of non-successful recanalization. Acquiring ASL with a single PLD provides lower CBF estimates in ischemic core compared to ASL with multiple PLDs in recanalized patients, especially when ATT is substantially shortened.

**Data availability**

The data that support the findings of this study are available from the CONTRAST Data access and writing committee (dawc.contrast@contrast-consortium.nl). Analytical methods such as the R syntax and output files of the analyses will be made available on request from the corresponding author.

## Funding

This work is funded in part through the Collaboration for New Treatments of Acute Stroke (CONTRAST) consortium, which acknowledges the support from the Netherlands Cardiovascular Research Initiative, an initiative of the Dutch Heart Foundation (CVON2015-01: CONTRAST); and from the Brain Foundation Netherlands (HA2015.01.06). The collaboration project is additionally financed by the Ministry of Economic Affairs by means of the PPP Allowance made available by Top Sector Life Sciences & Health to stimulate public–private partnerships (LSHM17016). This work was further funded in part through unrestricted funding by Stryker, Medtronic, and Cerenovus. EW is funded by a “Veni Vernieuwingsimpuls” from the Dutch Research Council entitled “Food for thought: Oxygen delivery to the brain”, Grant No 91619121.

## Declaration of Competing Interest

The authors declare that they have no known competing financial interests or personal relationships that could have appeared to influence the work reported in this paper.

## Data Availability

Data will be made available on request.
