# Supplementary material for: Cerebral blood flow quantification with multi-delay arterial spin labeling in ischemic stroke and the association with early neurological outcome
Source: Neuroimage Clin. 2023 Jan 31;37:103340. doi: 10.1016/j.nicl.2023.103340 (PMC9932490; doi:10.1016/j.nicl.2023.103340)
Supplement: Supplementary data 1 [file mmc1.docx]

**Supplementary Material**

**
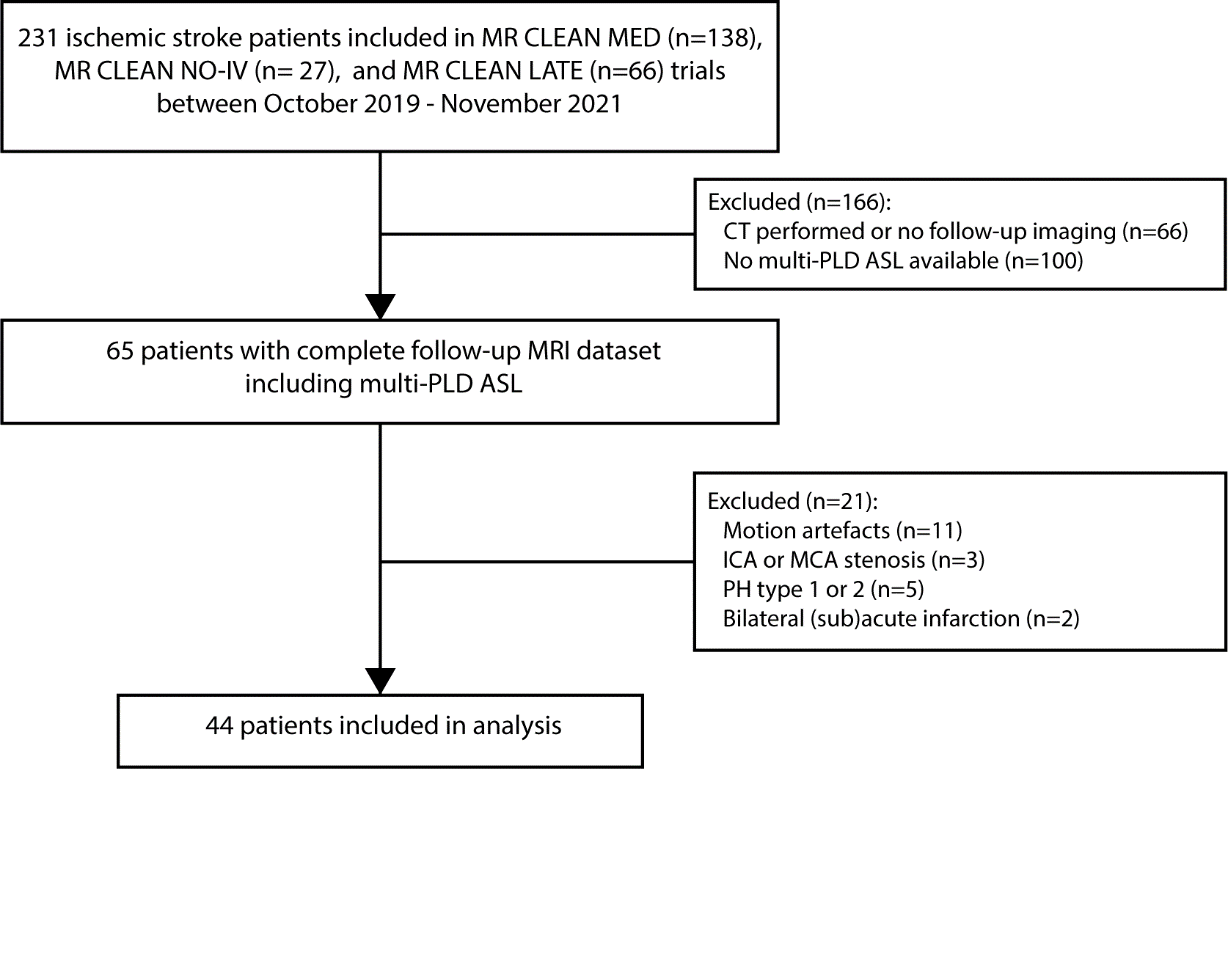
**

**supplementary figure 1.** Patient flowchart

**supplementary table 1.** Mean CBF values (ml/100g/min) when using 7 PLDs and images acquired at a PLD of 1.8s

|  | **Mean CBF±SD**  **7 PLDs** | **Mean CBF±SD**  **1.8s PLD** | **p-value** |
| --- | --- | --- | --- |
| **Recanalized (n=37)** |  |  |  |
| Ischemic core | 84.8 ± 33.0 | 78.6 ± 25.0 | 0.04 |
| Salvaged Penumbra* | 55.0 ± 20.3 | 56.7 ± 17.2 | 0.47 |
| Normal brain | 53.7 ± 15.1 | 51.2 ± 13.4 | 0.15 |
| GM | 49.6 ± 16.8 | 50.1 ± 13.8 | 0.70 |
| **Non-recanalized (n=7)** |  |  |  |
| Ischemic core | 16.5 ± 9.0 | 20.0 ± 7.0 | 0.21 |
| Normal brain | 48.4 ± 23.1 | 48.0 ± 14.3 | 0.83 |
| GM | 44.5 ± 20.6 | 43.8 ± 13.1 | 0.93 |

Abbreviations: CBF, cerebral blood flow; PLD, post-labeling delay; GM, grey matter.
* Values in salvaged penumbra are derived from a subset of recanalized patients with baseline CTP available (n=18).
